# Fingerprint Analysis of *Cnidium monnieri* (L.) Cusson by High-Speed Counter-Current Chromatography

**DOI:** 10.3390/molecules24244496

**Published:** 2019-12-08

**Authors:** Xiaoxue Wu, Xuemin Gao, Xuan Zhu, Shuyi Zhang, Xinmei Liu, Huayu Yang, Hua Song, Qing Chen

**Affiliations:** Fujian Provincial Key Laboratory of Innovative Drug Target Research, School of Pharmaceutical Sciences, Xiamen University, Xiamen 361002, China; xiaoxue_wu1@126.com (X.W.); holygxm@xmu.edu.cn (X.G.); zhuxuan@xmu.edu.cn (X.Z.); shuyi_zhang95@163.com (S.Z.); liuxinmei6427@163.com (X.L.); huayu_yang@126.com (H.Y.)

**Keywords:** fingerprint, high-speed counter-current chromatography (HSCCC), *Cnidium monnieri* (L.) Cusson, traditional Chinese medicine (TCM), quality control

## Abstract

*Cnidium monnieri* (L.) Cusson is a popular Traditional Chinese Medicine (TCM) with a variety of bioactivities. However, there are some problems that have affected the development of *Cnidium monnieri* (L.) Cusson. At present, many methods have been reported for the analysis of coumarins in *Cnidium monnieri* (L.) Cusson. However, the quality control of coumarins in *Cnidium monnieri* (L.) Cusson by high-speed counter-current chromatography (HSCCC) has not been reported. In this study, analytical high-speed counter-current chromatography (HSCCC) was successfully used for fingerprint of *Cnidium monnieri* (L.) Cusson with a two-phase solvent system composed of *n*-hexane-ethyl acetate-methanol-water at 4:6:6.5:3.5 (*v/v*). The UV wavelength was set at 254 nm. Six coumarin compounds with high biological activity were selected as indicator compounds for the quality control. The HSCCC fingerprint of the *Cnidium monnieri* (L.) Cusson was successfully established and there were some differences according to the results of the fingerprint analysis. The present results demonstrate that HSCCC is an established and efficient technique for the fingerprint analysis of *Cnidium monnieri* (L.) Cusson and can be used to control the quality of *Cnidium monnieri* (L.) Cusson. In brief, HSCCC is a useful technology for the fingerprint analytical method for TCM.

## 1. Introduction

**Figure 1:**
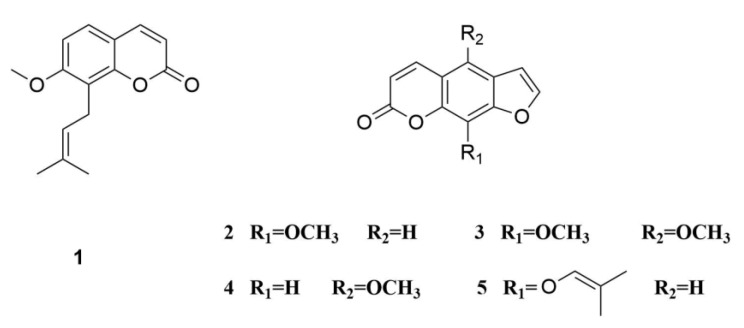
The structure of coumarin compounds of *Cnidium monnieri* (L.) Cusson. **1**, osthole; **2**, xanthotoxin; **3**, isopimpinellin; **4**, bergapten; **5**, imperatorin.

*Cnidium monnieri* (L.) Cusson is a useful traditional Chinese medicine (TCM). It is located in all parts of China, and is also distributed throughout the Russian Federation, North Korea, Vietnam, North America, and some European countries. Coumarins are the principal active ingredients, including osthole (1), xanthotoxin (2), isopimpinellin (3), bergapten (4), and imperatorin (5) (Figure 1) [1]. These compounds are applied in various fields such as agriculture application: skilling worms and drying dampness; and medical application: relieving asthma, increasing sperm production, and expelling cold and rheumatism [2]. Different pharmacological activities such as antiallergic, antipruritic, antidermatophytic, antibacterial, antifungal, and antiosteoporotic activities have been found for these coumarins on the basis of modern pharmacological studies [3,4]. Osthole is a selective antiproliferative agent in vascular smooth muscle cells [5] and causes hypotension in vivo; it also inhibits platelet aggregation and smooth muscle contraction in vitro. Osthole may also interfere with calcium influx and with cyclic nucleotide phosphodiesterases [6]. Bergapten possesses anti-inflammatory and analgesic activities [7] while imperatorin exhibits strong cytotoxic activity on human leukemia cells, as well as chemopreventive effects on hepatitis and skin tumors and anti-inflammatory activity [8,9,10].

The main chemical components of *Cnidium monnieri* (L.) Cusson are often unstable because of different origins and harvesting times, so it is difficult to evaluate its quality directly from its appearance. At the same time, because of the high profits in the TCM market, fraud has always existed, such as mixing spurious with genuine sources, trumpeting the curative effect of TCM. It is difficult to distinguish between fake and shoddy drugs. Many counterfeit drugs have been discovered on the market, for example *Apium graveoleus* L.var.dulce DC and *Lappula myosotis* V. Wolf. They are similar to *Cnidium monnieri* (L.) Cusson in terms of their morphological characteristics. These problems of quality control in TCM need to be solved through a new, unique, and effective method. At present, the fingerprint technique is an important method and widely used for the quality control of TCM.

Many analytical methods have been used in the study of *Cnidium monnieri* (L.) Cusson, such as gas chromatography (GC), thin layer chromatography (TLC), and high-performance liquid chromatography (HPLC). The *Cnidium monnieri* (L.) Cusson mainly contains coumarins such as osthol, imperatorin, and other coumarins, therefore the analysis of coumarins is of great significance in controlling the quality of *Cnidium monnieri* (L.) Cusson.

The TCM fingerprint is a chromatogram or spectrogram that can define the chemical characteristics of a TCM preparation by analytical means after proper processing [11]. The type and quantity of chemical components can be assessed comprehensively. Moreover, a more useful evaluation system can be applied in TCM by effectively reflecting the integrity and comprehensive function of Chinese medicinal ingredients. Many methods are used in the study of fingerprint, including chromatography methods [12], spectroscopic methods, and other methods, such as high performance liquid chromatography (HPLC) [13], UV spectroscopy (UV) [14], IR spectrum (IR) [15], and mass spectrometry (MS) [16]. Among them, HPLC is mostly applied to fingerprint analysis. HPLC is characterized by high separation efficiency, high selectivity, and wide application range. However, the sample needs strict pretreatment and it is difficult to analyze high viscosity samples, which are easy to cause fixed phase adsorption. Therefore, it is necessary to develop a fast and accurate method that can overcome the shortcomings of HPLC method for the quality control of TCM.

High-speed counter-current chromatography (HSCCC) offers a different mode of operation to conventional processes. It is a support-free liquid–liquid partition chromatography method, invented in the early 1970s by Ito [17]. HSCCC has a number of advantages, for instance no irreversible adsorption, acceptable efficiency, ease of scaling up, low risk of sample denaturation, minimal tailing of peaks, high recovery, the ability to accept particulates, and low solvent consumption compared with traditional separation methods. HSCCC technology has been widely used as a separation method for TCM components. It is proven that many coumarins can be isolated by HSCCC from different plants, such as apigenin and *d*-laserpitin [18,19,20]. Therefore, it is clear that HSCCC plays a non-substitutable role in the separation and analysis of coumarins. In this study, HSCCC was successfully applied in the fingerprint study of *Cnidium monnieri* (L.) Cusson. The main chemical constituents of *Cnidium monnieri* (L.) Cusson were compared in different batches and different origins. The HSCCC fingerprint of *Cnidium monnieri* (L.) Cusson was established, and a comprehensive quality evaluation of similarity was calculated, which provided a reliable basis for controlling the quality of *Cnidium monnieri* (L.) Cusson. The HSCCC method can also be extended to the application of other TCM products.

## 2. Results and Discussion

### 2.1. Selection of Extraction Methods

The results of the three extraction methods were as follows: It was revealed that 95% ethanol extraction (leaching and ultrasonic extraction) was obviously superior to the traditional decoction method as shown in Table 1, in which the total contents of osthole and imperatorin of the total coumarins of *Cnidium monnieri* (L.) Cusson were nearly 10 times higher than those of the latter. Considering the chemical structure and physicochemical properties of coumarins, the stability of traditional decoction was seriously affected by the high temperature. Compared with ultrasonic extraction, the extraction rate of imperatorin was increased by ethanol leaching, but the operation time was too long, and the extraction rate of osthole was lower than with ultrasonic extraction. Therefore, ultrasonic extraction was selected for the extraction of *Cnidium monnieri* (L.) Cusson.

### 2.2. Optimization of the Extraction Conditions

According to the R value of the orthogonal experimental results in Table 2 and Table 3, the order of factors affecting the extraction efficiency of imperatorin was A > B > C; ethanol concentration was the most important. The order of factors affecting the extraction efficiency of osthole was A > C > B, i.e., the ethanol concentration was the most important, and the ultrasonic extraction time was the second most important. Further analysis of variance revealed that the ethanol concentration and ultrasonic extraction time had a significant effect on osthole and imperatorin extraction (*p* < 0.01). According to the *K*-value of Table 3, the best condition for ultrasonic extraction was A_2_B_3_C_2_. The optimum extraction conditions of total coumarins were verified by repeated experiments. The concentrations of osthole and imperatorin were 7.64 and 8.43 μg*mL^−1^, respectively, using the optimum extraction conditions. The condition A_2_B_3_C_2_ was selected for the extraction of *Cnidium monnieri* (L.) Cusson.

### 2.3. Optimization of HSCCC Conditions

**Figure 2:**
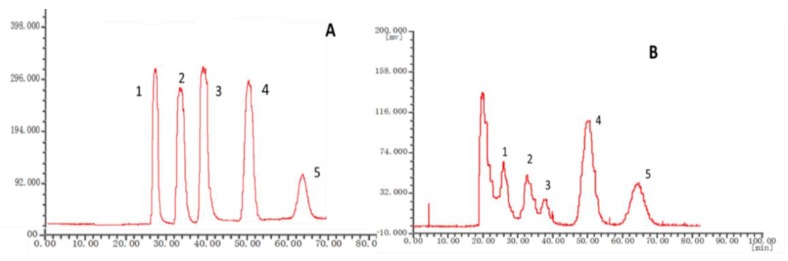
(**A**) HSCCC chromatogram of standard compounds. (**B**) HSCCC chromatogram of the crude sample of *Cnidium monnieri* (L) Cusson. **1**: xanthotoxin **2**: isopimpinellin **3**: bergapten **4**: imperatorin **5**: osthole. Condition: Solvent system: *n*-hexane-ethyl acetate-methanol-water: 4:6:6.5:3.5; speed: 1300 rpm; injection volume: 3 mg; flow rate: 1.0 mL/min.

Several HSCCC parameters were examined to ensure the separation of the compounds. Different flow rates, revolution speeds, detection wavelengths, and sample sizes were tested. For the separation of the crude extract, the flow rate, revolution speed, detection wavelength, and sample weight were set at 1.0 mL/min, 1300 rpm, 254 nm, and 3.0 mg. The main peaks of crude extract obtained baseline separation. Under these conditions, six fractions were produced (shown in Figure 2). (see Supplementary Materials).

### 2.4. Selection of the Two-Phase Solvent System

An appropriate two-phase solvent system plays an important role in HSCCC separation [21,22]. To select a suitable two-phase solvent system, previous reports on HSCCC were consulted, and some rules and characteristics of the target compounds were also considered (Hou et al., 2010). Previous literature has shown that the two-phase solvent system should satisfy the following requirements: (i) the *K*-values of the target compounds should be between 0.2 and 5; (ii) higher retention of the stationary phase provides better peak resolution; (iii) a short settling time (<30 s) of two-phase solvent system is important for the retention of the stationary phase; (iv) to separate two compounds, the ratio of their *K*-values or the separation factor (α = *K*_1_/*K*_2_, where *K*_1_ > *K*_2_) should be greater than 1.5.

Most of coumarins were medium polarity compounds, for which commonly used solvent systems in HSCCC are hexane/ethyl acetate/methanol/water. Thus, taking polarity into account, the following two-phase solvent systems(see Supplementary Materials) were tested with different ratios of the solvent system including *n*-hexane-ethyl acetate-methanol-water (5:5:5:5, 5:5:6:4, 5:5:6.5:3.5, 6:4:6.5:3.5, 4:6:6.5:3.5, *v/v/v/v*). The results are summarized in Table 4.

The *K*-values of the six peaks in the crude sample are presented in Table 4. In the tested biphasic solvent systems, *n*-hexane-ethyl acetate-methanol-water (5:5:5:5) and *n*-hexane-ethyl acetate-methanol-water (5:5:6:4) were rejected, as they displayed *K*-values greater than 2. The *K*-values of the solvent systems *n*-hexane-ethyl acetate-methanol-water (5:5:6.5:3.5) and *n*-hexane-ethyl acetate-methanol-water (6:4:6.5:3.5) were less than 0.5, and thus were unreasonable for application in whole HSCCC separation. Therefore, the solvent system *n*-hexane-ethyl acetate-methanol-water with the volume ratio of 4:6:6.5:3.5 was finally chosen to isolate the target compounds in the HSCCC analysis. Although the *K*-values of peak 1, 3, and 4 in the solvent systems *n*-hexane-ethyl acetate-methanol-water (4:6:6.5:3.5) are close, the content and efficacy of the whole active ingredient are mainly considered in the fingerprint of traditional Chinese medicine. Therefore, even if the separation degree of these peaks is slightly worse, it does not affect the application of high-speed countercurrent chromatography to the fingerprint analysis of *Cnidium monnieri* (L.) Cusson. Above all, the solvent system *n*-hexane-ethyl acetate-methanol-water with the volume ratio of 4:6:6.5:3.5 was finally chosen to isolate the target compounds in the HSCCC analysis.

### 2.5. Methodological Verification

Table 5 demonstrates the RSD of each peak in six samples of the same batch of *Cnidium monnieri* (L.) Cusson analyzed by HSCCC. The RSD of the relative peak areas of each compound was less than 2.64%, which indicated that good reproducibility was shown in this method. After assessing the same sample solution six times continuously and calculating its relative value, the results show that the RSD of the relative peak area ranged from 1.75% to 2.84%, which demonstrated that the precision was good. The RSD of the relative peak area after 24 h is displayed in Table 5. The relative peak area of RSD was less than 3.00%, indicating that the sample was stable within 24 h. All the data illustrated that this reliable, accurate, and reproducible method is suitable for the fingerprint study of *Cnidium monnieri* (L.) Cusson (see Supplementary Materials).

### 2.6. Fingerprint of HSCCC

**Figure 3:**
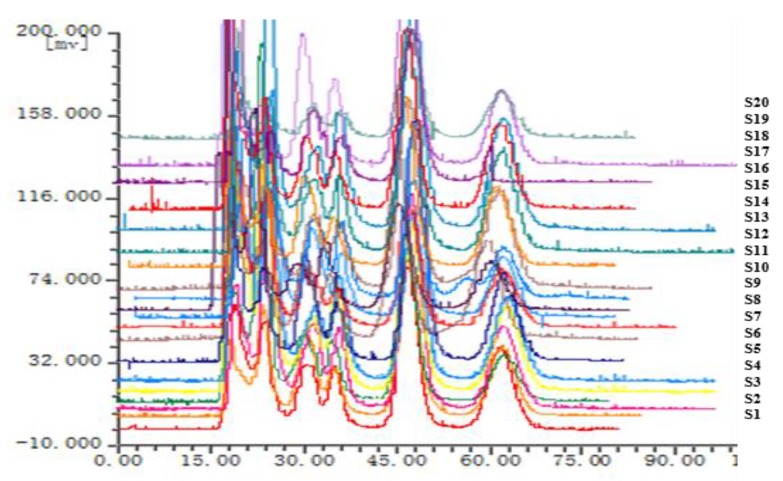
The fingerprint of *Cnidium monnieri* (L.) Cusson. S1–S20 are the fingerprints of 20 batches.

**Figure 4:**
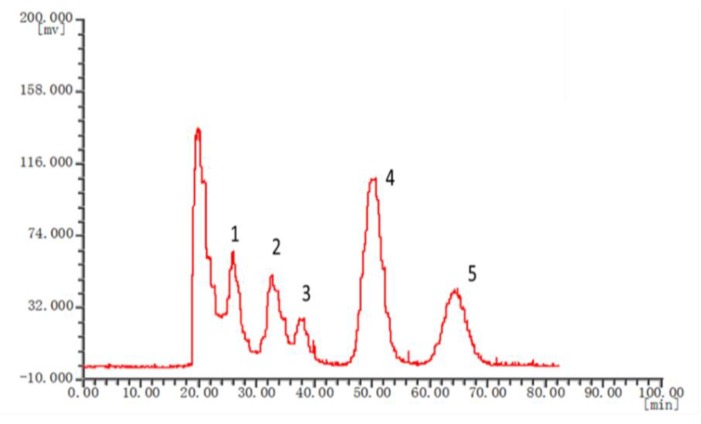
Fitting curve of the fingerprint of *Cnidium monnieri* (L.) Cusson. **1**: xanthotoxin **2**: isopimpinellin **3**: bergapten **4**: imperatorin **5**: osthole. Condition: Solvent system: *n*-hexane-ethyl acetate-methanol-water: 4:6:6.5:3.5; speed: 1300 rpm; injection volume: 3 mg; flow rate: 1.0 mL/min.

**Figure 5:**
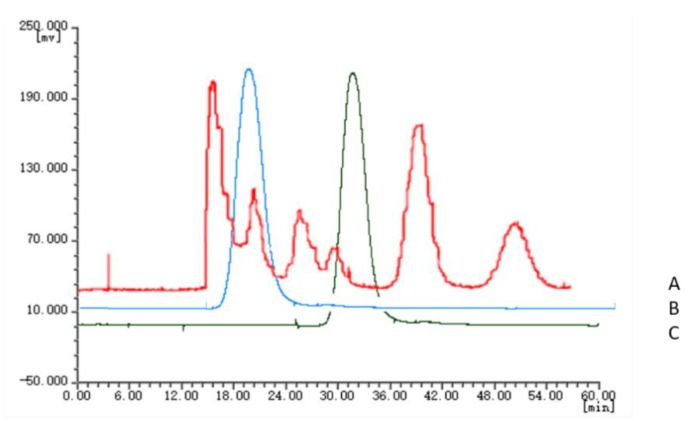
High-speed counter-current chromatography (HSCCC) chromatogram of *Cnidium monnieri* (L) Cusson, *Apium graveoleus* L.var.dulce DC and *Lappula myosotis* V. Wolf. **A**: *Cnidium monnieri* (L) Cusson; **B**: *Lappula myosotis* V. Wolf; **C**: *Apium graveoleus* L.var.dulce DC.

The chromatographic fingerprints of 20 batches of *Cnidium monnieri* (L.) Cusson were analyzed using the software “similarity evaluation system for chromatographic fingerprint of TCM (2004A)”. Fingerprints and similarity were generated after data processing (see Supplementary Materials). There were six common peaks in the fingerprint: peak 2 was identified based on its chemical composition as xanthotoxin, peak 3 was isopimpinellin, peak 4 was bergapten, peak 5 was imperatorin, and peak 6 was osthole. As a result of the largest peak area displayed in Figure 2 was peak 5, the peak area of peak 5 in the 20 batches samples was set to 1, and the other peak areas were expressed as relative values (Table 6). The fingerprint analysis of *Cnidium monnieri* (L.) Cusson is exhibited in Figure 3 and Figure 4. The similarity of the fingerprints of *Cnidium monnieri* (L.) Cusson was calculated and is shown in Table 7. The similarity calculation results show that the similarity of 20 batches of *Cnidium monnieri* (L.) Cusson fingerprint was greater than 0.80, manifesting that there were some differences between them. The chromatograms of *Cnidium monnieri* (L.) Cusson and its counterfeits are shown in Figure 5. *Cnidium monnieri* (L.) Cusson can be distinguished from its counterfeits, as can be seen in Figure 5.

## 3. Discussion

*Cnidium monnieri* (L.) Cusson is a useful traditional Chinese herb. Extraction efficiency of *Cnidium monnieri* (L.) Cusson can be influenced by different solvents. The total coumarins of *Cnidium monnieri* (L.) Cusson have good solubility in ethanol and the ethanol extract has obvious pharmacological effects. Therefore, ethanol is often used for the extraction and separation of total coumarins from *Cnidium monnieri* (L.) Cusson. Previously used extraction methods of coumarin from *Cnidium monnieri* (L.) Cusson include cold maceration, water decoction, and ultrasonic methods. In this study, based on the comprehensive comparison and quantitative analysis of the differences in the content of osthole and imperatorin extracted by the ultrasonic method, ethanol leaching, and decoction extraction, the superiority of ultrasonic technology was clarified in the extraction of total coumarins from *Cnidium monnieri* (L.) Cusson. Different extraction conditions also affected the efficiency of ultrasonic extraction, such as the concentration of the solvent, the volume of the solvent, and ultrasound duration. Three factors affecting the extraction rate of osthole and imperatorin in ultrasonic extraction technology were selected for this assessment, and orthogonal table L_9_ (3^3^) was used to optimize the extraction conditions of total coumarins from *Cnidium monnieri* (L.) Cusson. The results discover that the optimum conditions for extracting total coumarins from *Cnidium monnieri* (L.) Cusson were 3 mg powder dissolved in 40 mL of 75% ethanol and subjected to ultrasonic extraction for 40 min.

The fingerprint of TCM provides a quality control index for the determination of a single component as well as the internal quality of the whole preparation, with this, a comprehensive evaluation of the internal quality and comprehensive control for all TCM preparations is now possible. More comprehensive information is provided by fingerprint than the determination of one component. In this experiment, 20 batches of *Cnidium monnieri* (L.) Cusson samples were tested. An analytical method for fingerprint of *Cnidium monnieri* (L.) Cusson by HSCCC was established. The similarities and differences in the chemical composition of different batches and the origins of medicinal materials were qualitatively analyzed according to the similarity evaluation results obtained by HSCCC, which comprehensively and reliably reflected the quality of *Cnidium monnieri* (L.) Cusson. The experimental results indicate that the fingerprint constructed using the optimum chromatographic conditions can reveal the intrinsic characteristics of *Cnidium monnieri* (L.) Cusson with high similarity obtained between batches. Therefore, HSCCC chromatographic technology can be used for the quality control of *Cnidium monnieri* (L.) Cusson. As can be seen in Figure 2, peak 2, xanthotoxin was selected as a reference because of the good peak separation and shape, and the relative peak areas of the other common peaks were calculated. Distinctive morphological characteristics of the retention time, peak shape, peak height and peaks characteristics were shown in the peaks of *Cnidium monnieri* (L.) Cusson, which can be easily distinguished from counterfeits such that *Cnidium monnieri* (L.) Cusson can be identified. Some peak areas are displayed in Table 6, and it can be seen that the same peaks were common in the 20 batches of this medicinal materials; these similarities are also exhibited in Table 7 and Figure 3. These results revealed that differences in the same medicinal material from different batches and origins can be distinguished by our method. Therefore, the origin and batch affect the chemical composition of medicinal materials, and fingerprints with some differences were found in the 20 batches of medicinal material. The fingerprints of *Cnidium monnieri* (L.) Cusson were similar to each other in terms of geographic location, which indicates that the quality of *Cnidium monnieri* (L.) Cusson can be affected by geographical and climatic conditions. There were large differences in the fingerprints of *Cnidium monnieri* (L.) Cusson from the same origins and different batches, which illustrates that processing also has some influence on the quality of *Cnidium monnieri* (L.) Cusson. It is suggested that the origins and batches of medicinal materials be considered in order to ensure that TCM can be identified and the quality can be differentiated.

Because of their similar morphological characteristics with *Cnidium monnieri* (L.) Cusson, many counterfeit drugs such as *Apium graveoleus* L.var.dulce DC and *Lappula myosotis* V. Wolf are found on the market. This uncertain quality is difficult to identify by ordinary people. However, different chemical compositions and pharmacological activities are known to exist in TCM. The HSCCC chromatograms of three medicinal materials are shown in Figure 5. These three medicinal materials can be clearly distinguished using the chromatograms. Therefore, *Cnidium monnieri* (L.) Cusson can be easily discriminated using the HSCCC method.

In this study, at first, the samples were assessed without preliminary extraction before HSCCC. HSCCC is suitable for complex samples such as TCM preparations and biological products. There are many complex compounds in TCM, which will stain chromatography columns. HSCCC is a support-free liquid–liquid partition chromatography method, which does not cause reversible adsorption. Moreover, the high resolution and reproducibility of HPLC and HSCCC are similar, so HSCCC is suitable for fingerprint of TCM, as it can be considered equivalent to HPLC. However, HSCCC analysis has advantages regarding costs. The cost of the HSCCC instrument and required reagents are lower than with HPLC. Thus, HSCCC is an inexpensive, reliable, cost effective, and easy to operate method, and represents a modern technology for the quality control of TCM.

## 4. Materials and Methods

### 4.1. Apparatus

The fruits of *Cnidium monnieri* (L.) Cusson were ground into a fine powder by using a multifunctional crusher (Jinsui Machinery Manufacturer, Dalian, China, 28,000 rpm/min). Ultraviolet spectrophotometry employed a silicon light diode, deuterium lamp, and tungsten lamp (SP-752, Shanghai Spectroscopy Instrument Co., Ltd., Shanghai, China). The wavelength range was set at 190–900 nm. The measured wavelength of osthole was set at 245 nm and imperatorin was at 301 nm.

The high-performance liquid chromatography (HPLC) equipment was a Shimadzu LC-10A system. It was used to measure the partition coefficients (*K*-value). The HPLC analysis was consisted of a Kromasil C_18_ column (250 × 4.6 mm id, 5 µm, Akzo Nobel). The mobile phase was composed of water (A) and methanol (B). A gradient programmer was used according to the following profile: 0 min 40% B; 40 min 60% B. The flow rate was 1.0 mL/min at 25 °C. The chromatograms were recorded at 245 nm.

HSCCC was performed using a model OptiChrome TM-30B analytical HSCCC (Counter Current Technology Co., Ltd., Beijing, China). The apparatus was equipped with two polytetrafluoroethylene analytical coils (diameter of tube, 2.6 mm; total volume, 30 mL) and a 1 mL manual sample loop. The rotational speed of the apparatus can be regulated with a speed controller in the range between 0 and 1500 rpm. The effluent was detected online at 254 nm with a Model UVD-680-4 detector (Jinda Biotech, Shanghai, China) and a model of V4.0 work station (Jinda Biotech, Shanghai, China).

### 4.2. Reagents and Materials

All solvents used for preparation of crude samples and HSCCC separation were of analytical grade (Sinopharm Chemical Reagents, Shanghai, China). Methanol used for HPLC analysis was of chromatographic grade and was purchased from Sigma (USA). Water used in this study was generated by a water purification system (RSJ Scientific Instruments Co. Xiamen China). *Lappula myosotis* V. Wolf and *Apium graveoleus* L.var.dulce DC were purchased from a Deyongtang drugstore (Anhui, China). Twenty batches of *Cnidium monnieri* (L.) Cusson were purchased from different pharmacies (Table 8). Osthole, xanthotoxin, isopimpinellin, bergapten and imperatorin (purity > 98%) were purchased from Nanjing Jingzhu Biological Science and Technology Co., Ltd.

### 4.3. Selection of Extraction Conditions for Cnidium monnieri (L.) Cusson

Osthole, xanthotoxin, isopimpinellin, bergapten, and imperatorin were precisely weighed and prepared into 1 mg*mL^−1^ of solutions with methanol. Then, a certain amount of each standard solution was absorbed and diluted with methanol to obtain a series of solutions with different concentrations.

Osthole and imperatorin were the major coumarin-type compounds, and other compounds in *Cnidium monnieri* (L.) Cusson were similar to imperatorin in structure. As a result, osthole and imperatorin were selected to represent the main coumarins of *Cnidium monnieri* (L.) Cusson and were used for the measurement of the main coumarins. The fruits of *Cnidium monnieri* (L.) Cusson were ground into fine powder and dried for 6 h at 25 °C, then were stored in a dryer. Before analysis, 3.0 g of the power was weighed precisely, and the following extraction processes were used: (1) the extract was collected by adding 20 mL of 95% ethanol at room temperature for 24 h; (2) 20 mL of distilled water was added for heating and decocting for 30 min; (3) ultrasonic extraction was performed for 30 min after adding 20 mL of 95% ethanol. The extracted solutions were filtered through filter paper and concentrated at 55 °C to dry separately. Finally, the contents of osthole and imperatorin in the total coumarins of *Cnidium monnieri* (L.) Cusson were determined by different extraction methods using an ultraviolet spectrophotometer.

### 4.4. Orthogonal Design Optimization of Ultrasonic Extraction Conditions

According to the results of the different extraction methods, three factors affecting the ultrasonic extraction rate of osthole and imperatorin were selected (ethanol concentration (A), ethanol volume (B), and ultrasonic time (C)) to optimize the extraction conditions of total coumarins from *Cnidium monnieri* (L.) Cusson. The orthogonal design was applied in the study and each factor was selected at three levels. The L_9_ (3^3^) orthogonal table was used to arrange the experiment. The specific influencing factors and the experimental levels were shown in Table 9.

3 g of *Cnidium monnieri* (L.) Cusson powder was added in a 100 mL conical bottle, then different proportions and volumes of ethanol were added and ultrasonic extraction was performed for different periods of time. The extracts of total coumarins from *Cnidium monnieri* (L.) Cusson were obtained by vacuum filtration and concentrated at 55 °C to dryness. The contents of osthole and imperatorin in the total coumarins were determined by ultraviolet spectrophotometer (according to Lambert-beer law). The operations were repeated three times in parallel.

A = abc (a: absorption coefficient; b: thickness of cuvette; c: the concentration of solution)

### 4.5. Preparation of the Crude Extract from Cnidium monnieri (L.) Cusson

The fruits of *Cnidium monnieri (L.)* Cusson were ground into a fine powder. Then, 9 g of the powder was weighted accurately and immersed in 120 mL of 75% methanol for half an hour in a conical flask. After ultrasonication for 40 min, it was cooled to room temperature and then filtered through filter paper. This extraction was repeated three times. The same extraction method was applied in the study of *Apium graveoleus* L.var.dulce DC and *Lappula myosotis* V. Wolf. The extracted solutions were combined and concentrated at 55 °C to dryness. Finally, the extracts were dissolved in methanol, dried and stored at 4 °C.

### 4.6. Selection of the HSCCC Solvent System

Successful separation of coumarins by HSCCC largely depends on the selection of a suitable two-phase solvent system according to the partition coefficients (0.5 < *K* < 2.0), which were determined by HPLC. Solutions with different ratios of *n*-hexane-ethyl acetate-methanol-water were prepared and thoroughly equilibrated in a separation funnel at room temperature. The measurement of *K*-values was performed as follows: 3 mg of the sample was dissolved in 2 mL of the upper phase and 2 mL of the lower phase of the preequilibrated two-phase solvent system. After shaking thoroughly, the two phases separated. A total of 2 mL of each phase was transferred into another centrifuge tube and filtered through a 0.45 µm membrane filter unit. Each sample solution (20 μL) was analyzed by HPLC. The *K*-value was defined as the ratio of the peak area of the compound in the upper phase divided by that in the lower phase.

### 4.7. Preparation of the Two-Phase Solvent System and Sample Solution

For the present study, a two-phase solvent system composed of different ratios of *n*-hexane-ethyl acetate-methanol-water (5:5:5:5, 5:5:6:4, 5:5:6.5:3.5, 6:4:6.5:3.5, 4:6:6.5:3.5, *v/v/v/v*) were selected for HSCCC. The solvent system was thoroughly equilibrated in a separation funnel at room temperature by repeated vigorous shaking and was allowed to settle overnight to avoid emulsification. Then, the upper phase and the lower phase were separated and degassed by ultrasound for 30 min before use. The upper phase was used as the stationary phase and the lower phase was used as the mobile phase. In the HSCCC separation, the sample solution was prepared by dissolving 3 mg of the sample in 1 mL of the mixture solution consisting of equal volumes of both the upper phase and lower phase (1:1, *v/v*). The standard samples solutions were prepared by dissolving 3 mg of standard product in 1 mL of the mixture consisting of equal volumes of the lower phase and upper phase of the solvent system.

### 4.8. Methodological Verification

#### 4.8.1. Reproducibility

Six crude extracts from the same batch of *Cnidium monnieri* (L.) Cusson were assessed according to the chromatographic conditions in Section 2.1 by HPLC. Then, the relative standard deviation (RSD) values of the six compounds were calculated.

#### 4.8.2. Precision

According to the chromatographic conditions in Section 2.1, the crude extract of *Cnidium monnieri* (L.) Cusson from the same batch was assessed six times by HPLC. The peak areas of the six compounds were recorded, and the RSD values were calculated.

#### 4.8.3. Stability

A batch of crude extract of *Cnidium monnieri* (L.) Cusson was obtained, and the peak areas of xanthotoxin, isopimpinellin, bergapten, imperatorin, and osthole were recorded after 0, 2, 4, 6, 8, and 12 h under the conditions described in Section 2.1 by HPLC, and the RSD values were calculated.

### 4.9. HSCCC Separation Procedure

For each separation, the upper phase (stationary phase) was filled up at a flow rate of 30 mL/min in the coiled HSCCC column at first, then the lower phase (mobile phase) was pumped into the outlet of the column at a flow rate of 1 mL/min and the rotator speed was set at 1300 rpm. After hydrodynamic equilibrium was established in the column, the sample solution (1 mL) containing 3 mg of the sample was injected through the injection valve. The effluent from the outlet of the column was continuously monitored at 254 nm by a UV detector (Figure 2B). After separation, the centrifuge was stopped and the solvents were pumped out from the column with pressurized air and collected in a graduated cylinder to measure the stationary phase retention volume. Twenty batches of *Cnidium monnieri* (L) Cusson and standard samples were analyzed by HSCCC. *Apium graveoleus* L.var.dulce DC and *Lappula myosotis* V. Wolf were also assessed under the same conditions. All separations were performed at room temperature (25 °C).

### 4.10. Fitting and Comparing the Fingerprint of Cnidium monnieri (L.) Cusson

The HSCCC chromatograms of 20 batches of *Cnidium monnieri* (L.) Cusson were analyzed by the software “similarity evaluation system for chromatographic fingerprint of TCM (2004A)”, which was issued by the National Pharmacopoeia Commission. Control fingerprints and similarity were generated after data processing.

## 5. Conclusions

HSCCC is widely used in the separation and preparation of TCM, but it was not popularized. Until now, the HSCCC method has only been applied in fingerprint studies on *Salvia miltiorrhiza* Bunge [23]. Semi-preparative HSCCC was used in that work, which took a long time. Nowadays, analytical HSCCC meets the needs of fingerprint studies and can be widely applied in the analysis of TCM. In this study, analytical HSCCC was successfully applied to fingerprint of *Cnidium monnieri* (L.) Cusson for the first time. The fingerprint of *Cnidium monnieri* (L.) Cusson was established by HSCCC and could easily distinguish the genuine product form counterfeits. This method can be easily used as the first step in quality control, i.e., identification. Next, the fitting curve of *Cnidium monnieri* (L.) Cusson was established, which can be used to identify the quality of *Cnidium monnieri* (L.) Cusson from different origins and batches. Many complex chemical constituents are present in TCM, and their quality can vary with different sources, batches, and origins. Therefore, this technique can be extended to the quality control of other TCM and provide quality assurance for TCM on the international market.

## Figures and Tables

**Table 1:** Effects of different extraction methods on the content of osthole and imperatorin in the total coumarins of *Cnidium monnieri* (L.) Cusson (n = 3).

Extraction Method	Content (mg*g^−1^)		
	Osthole	Imperatorin	Total
Ethanol leaching	22.22	9.85	32.06
Decoction extraction	3.19	0.56	3.75
Ethanol ultrasonic extraction	26.38	9.56	35.95

**Table 2:** L_9_ (3^3^) orthogonal test design and the concentrations of osthole and imperatorin (n = 3).

Test Number	A	B	C	(1) Osthole μg*mL^−1^	(2) Imperatorin μg*mL^−1^
1	1	1	1	2.78	0.91
2	1	2	2	2.84	3.47
3	1	3	3	3.01	5.61
4	2	1	1	4.83	6.94
5	2	2	2	3.15	5.18
6	2	3	3	6.49	6.33
7	3	1	1	3.23	4.50
8	3	2	2	3.08	3.73
9	3	3	3	3.40	4.62

**Table 3:** *K*-value and *R*-value at different conditions.

*K*-Value	A	B	C
*K*_1 (osthole)_	2.88	3.61	4.12
*K*_2 (osthole)_	4.82	3.02	3.69
*K*_3 (osthole)_	3.24	4.30	3.13
*R*_(osthole)_	1.95	1.28	0.99
*K*_1 (imperatorin)_	3.33	5.12	3.68
*K*_2 (imperatorin)_	7.15	4.13	6.01
*K*_3 (imperatorin)_	4.28	5.52	5.10
*R*_(imperatorin)_	3.82	1.39	2.35

*K*_i_: the sum of the test results corresponding to the horizontal number i on any column. *R*: *K*_Max_—*K*_Min_.

**Table 4:** Partition coefficient (K) of the target compounds measured in different solvent systems.

Solvent Systems (*v/v*)	*K*-Value					
	1	2	3	4	5	6
*n*-hexane-ethyl acetate-methanol-water (5:5:5:5)	1.52	0.57	1.09	1.62	4.63	7.17
*n*-hexane-ethyl acetate-methanol-water (5:5:6:4)	0.94	0.37	0.59	0.86	2.06	3.19
*n*-hexane-ethyl acetate-methanol-water (5:5:6.5:3.5)	0.87	0.12	0.28	0.45	1.07	1.78
*n*-hexane-ethyl acetate-methanol-water (6:4:6.5:3.5)	0.63	0.05	0.18	0.30	0.93	1.90
*n*-hexane-ethyl acetate-methanol-water (4:6:6.5:3.5)	0.92	0.67	0.92	0.90	1.49	1.99

**Table 5:** The reproducibility, precision, and stability of *Cnidium monnieri* (L.) Cusson.

Compound	Reproducibility	Precision	Stability
	RSD (%)	RSD (%)	RSD (%)
1	2.25	1.75	1.87
2	1.46	2.74	2.47
3	2.61	2.42	2.01
4	2.63	2.46	3.00
5	2.45	2.50	2.77
6	2.61	2.84	2.13

**Table 6:** Relative peak areas of *Cnidium monnieri* (L.) Cusson.

Number of Peaks	1	2	3	4	5	6
S1	0.11	0.16	0.16	0.12	1	0.47
S2	0.70	0.09	0.28	0.11	1	0.54
S3	0.40	1.05	0.24	0.18	1	0.59
S4	0.24	0.67	0.21	0.16	1	0.57
S5	0.83	0.70	0.16	0.25	1	0.54
S6	0.55	0.55	0.28	0.16	1	0.49
S7	0.66	0.13	0.24	0.10	1	0.48
S8	0.20	0.20	0.34	0.11	1	0.63
S9	0.58	0.21	0.32	0.19	1	0.58
S10	0.36	0.56	0.32	0.16	1	0.44
S11	0.64	0.72	0.16	0.30	1	0.51
S12	0.81	0.43	0.22	0.09	1	0.58
S13	0.20	0.27	0.25	0.11	1	0.56
S14	0.37	0.54	0.17	0.21	1	0.65
S15	0.43	0.22	0.17	0.13	1	0.62
S16	0.22	0.18	0.16	0.12	1	0.53
S17	0.44	0.04	0.13	0.09	1	0.58
S18	0.82	0.22	0.39	0.29	1	0.44
S19	0.34	0.12	0.23	0.13	1	0.67
S20	0.75	0.18	0.24	0.13	1	0.54

**Table 7:** The similarity of 20 batches of *Cnidium monnieri* (L.) Cusson.

Number	Similarity
S1	0.9
S2	0.86
S3	0.93
S4	0.92
S5	0.89
S6	0.93
S7	0.91
S8	0.92
S9	0.9
S10	0.93
S11	0.92
S12	0.88
S13	0.91
S14	0.95
S15	0.91
S16	0.9
S17	0.88
S18	0.83
S19	0.88
S20	0.84

**Table 8:** Sources and batch numbers of 20 batches of *Cnidium monnieri* (L.) Cusson.

Number	Source	Batch Number
1	Jiangsu	170,801
2	Anhui	170,524
3	Anhui	170,828
4	Anhui	161,109
5	Anhui	160,524
6	Zhejiang	150,810
7	Zhejiang	170,810
8	Zhejiang	160,910
9	Zhejiang	171,211
10	Hebei	170410
11	Hebei	160,805
12	Hebei	160,701
13	Hebei	140,301
14	Hebei	170,910
15	Hunan	170,309
16	Guangxi	170,807
17	Guangxi	170,410
18	Hubei	150,410
19	Jiangxi	171,105
20	Shangdong	171,112

**Table 9:** Factor table of ultrasonic extraction conditions.

Factor Level	A/%	B/mL	C/min
1	50	20	20
2	75	30	40
3	95	40	60

## Supplementary Materials

The following HSCCC procedure, methodological validation and fingerprint analysis are available as supporting data. Supplementary Materials are available online.
