# Supplementary material for: Fingerprint Analysis of Cnidium monnieri (L.) Cusson by High-Speed Counter-Current Chromatography
Source: Molecules. 2019 Dec 8;24(24):0. doi: 10.3390/molecules24244496 (PMC6969901; doi:10.3390/molecules24244496)
Supplement: Supplementary file 1 [file molecules-24-04496-s001.zip › supplementary material/Methodological verification.docx]

 Reproducibility

 Precision

Stability
