# Supplementary figures and images for: Fingerprint Analysis of Cnidium monnieri (L.) Cusson by High-Speed Counter-Current Chromatography
Source: Molecules. 2019 Dec 8;24(24):0. doi: 10.3390/molecules24244496 (PMC6969901; doi:10.3390/molecules24244496)

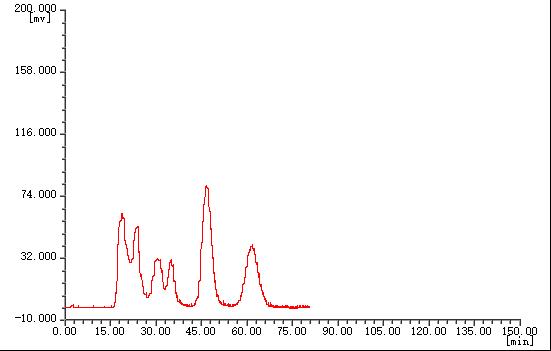

Supplement: Supplementary file 1 [file molecules-24-04496-s001.zip › supplementary material/medicinal materials/1.jpg]

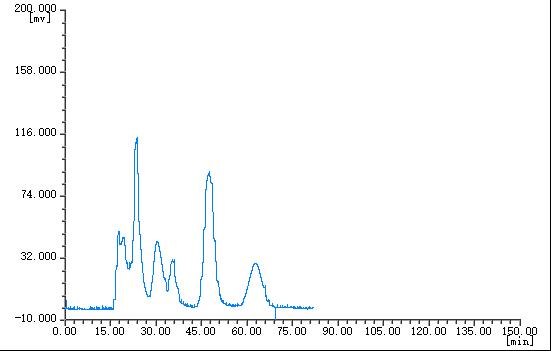

Supplement: Supplementary file 1 [file molecules-24-04496-s001.zip › supplementary material/medicinal materials/10.jpg]

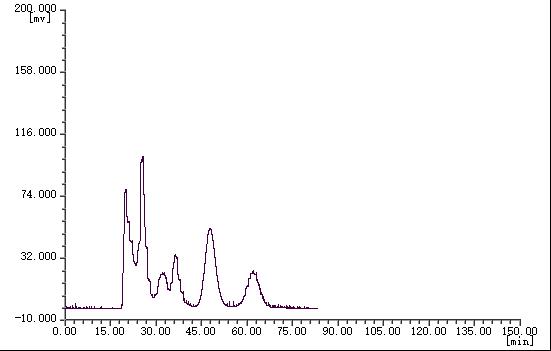

Supplement: Supplementary file 1 [file molecules-24-04496-s001.zip › supplementary material/medicinal materials/11.1.jpg]

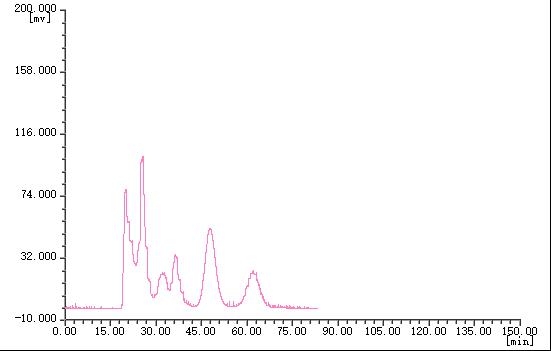

Supplement: Supplementary file 1 [file molecules-24-04496-s001.zip › supplementary material/medicinal materials/11.jpg]

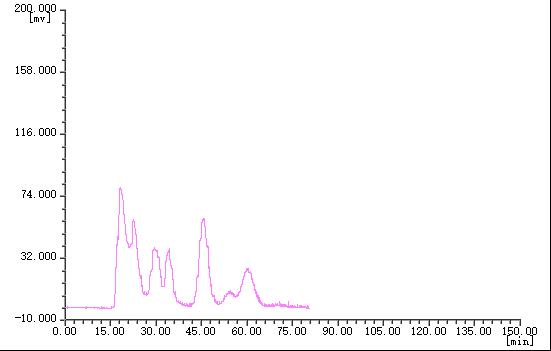

Supplement: Supplementary file 1 [file molecules-24-04496-s001.zip › supplementary material/medicinal materials/12.jpg]

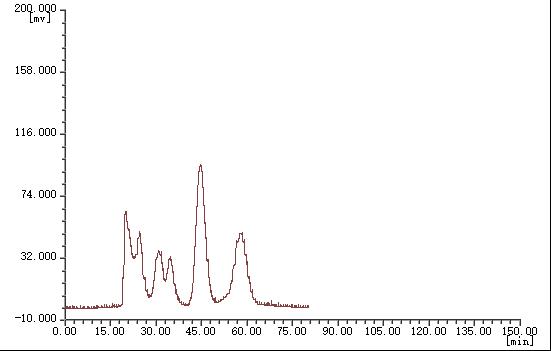

Supplement: Supplementary file 1 [file molecules-24-04496-s001.zip › supplementary material/medicinal materials/13.jpg]

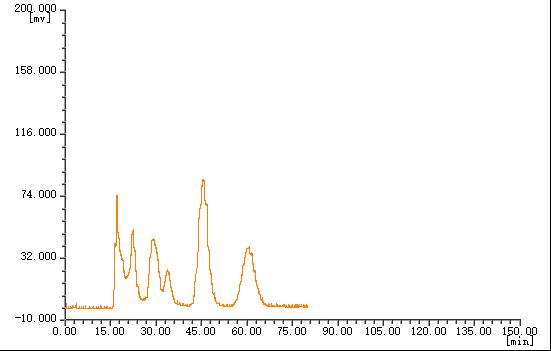

Supplement: Supplementary file 1 [file molecules-24-04496-s001.zip › supplementary material/medicinal materials/14.jpg]

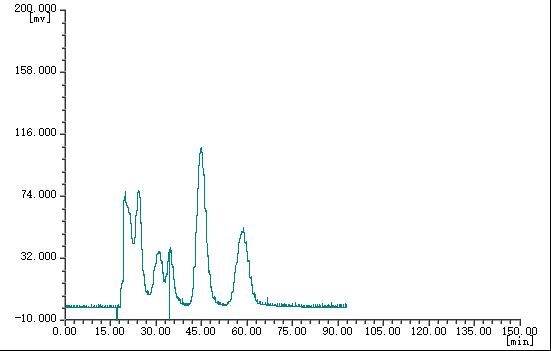

Supplement: Supplementary file 1 [file molecules-24-04496-s001.zip › supplementary material/medicinal materials/15.jpg]

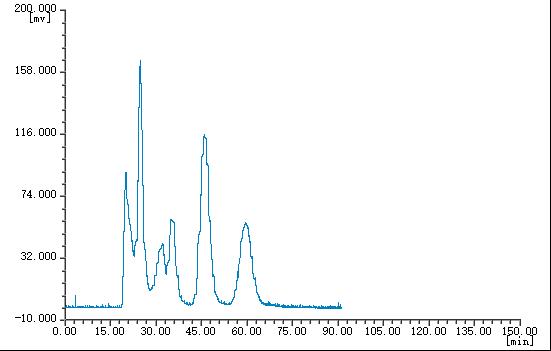

Supplement: Supplementary file 1 [file molecules-24-04496-s001.zip › supplementary material/medicinal materials/16.jpg]

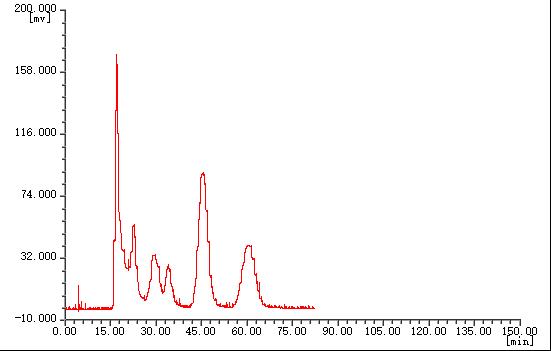

Supplement: Supplementary file 1 [file molecules-24-04496-s001.zip › supplementary material/medicinal materials/17.1.jpg]

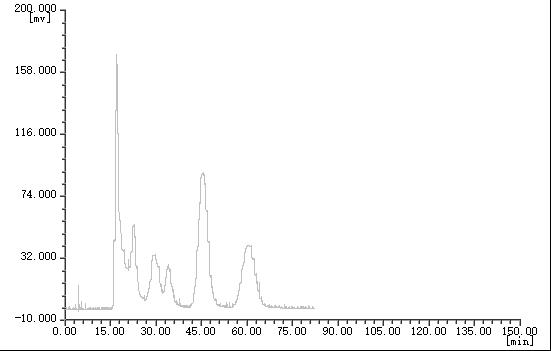

Supplement: Supplementary file 1 [file molecules-24-04496-s001.zip › supplementary material/medicinal materials/17.jpg]

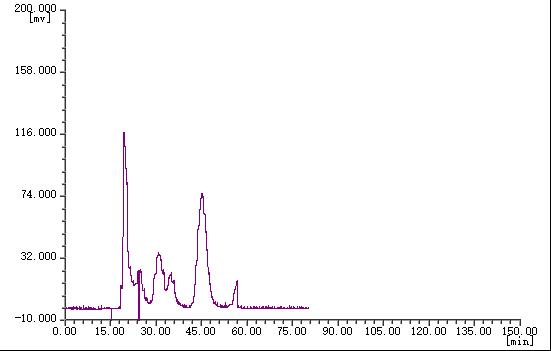

Supplement: Supplementary file 1 [file molecules-24-04496-s001.zip › supplementary material/medicinal materials/18.jpg]

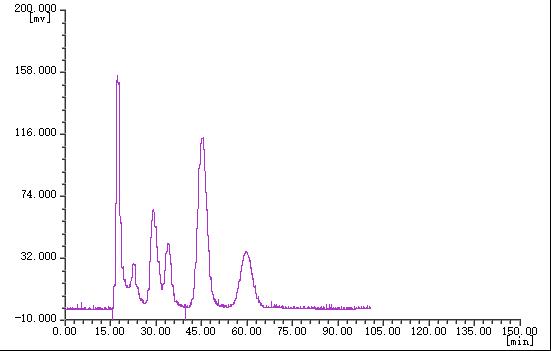

Supplement: Supplementary file 1 [file molecules-24-04496-s001.zip › supplementary material/medicinal materials/19.jpg]

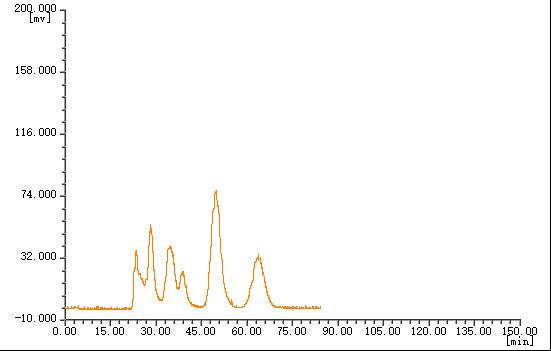

Supplement: Supplementary file 1 [file molecules-24-04496-s001.zip › supplementary material/medicinal materials/2.jpg]

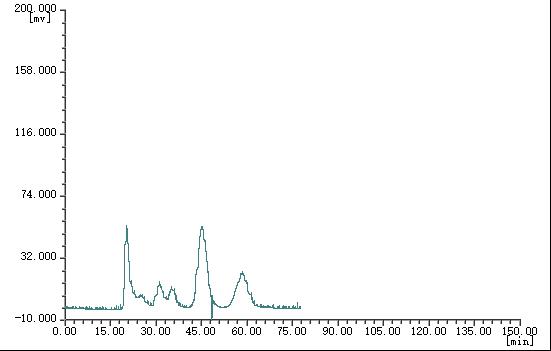

Supplement: Supplementary file 1 [file molecules-24-04496-s001.zip › supplementary material/medicinal materials/20.jpg]

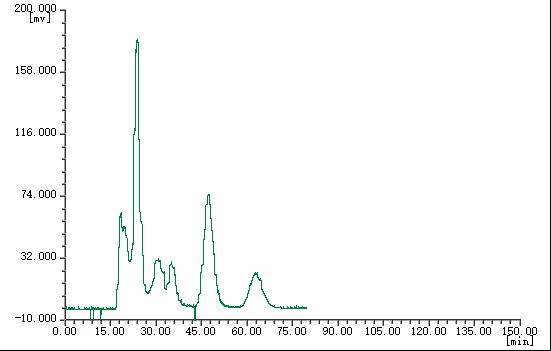

Supplement: Supplementary file 1 [file molecules-24-04496-s001.zip › supplementary material/medicinal materials/3.jpg]

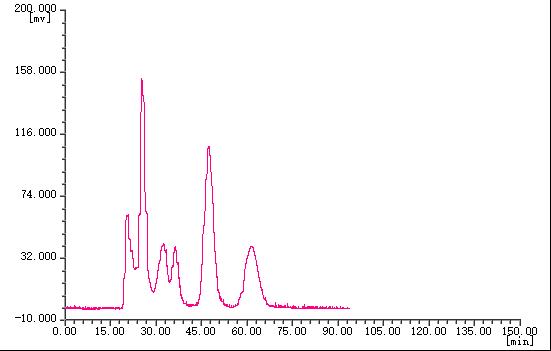

Supplement: Supplementary file 1 [file molecules-24-04496-s001.zip › supplementary material/medicinal materials/4.jpg]

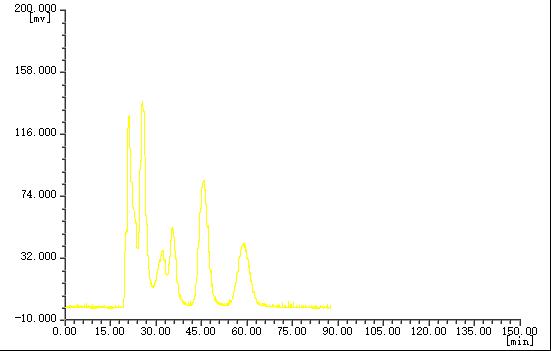

Supplement: Supplementary file 1 [file molecules-24-04496-s001.zip › supplementary material/medicinal materials/5.jpg]

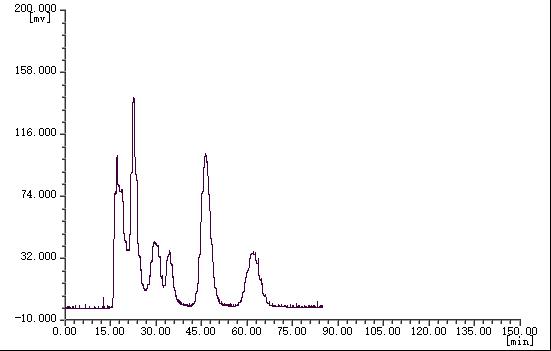

Supplement: Supplementary file 1 [file molecules-24-04496-s001.zip › supplementary material/medicinal materials/6.1.jpg]

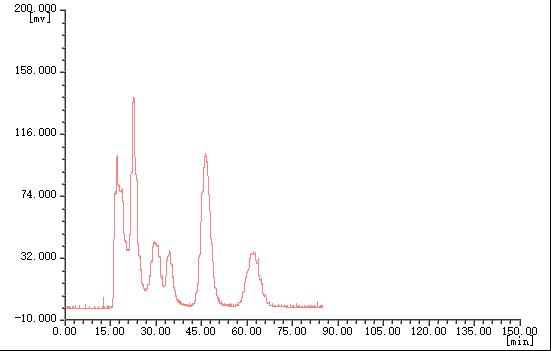

Supplement: Supplementary file 1 [file molecules-24-04496-s001.zip › supplementary material/medicinal materials/6.jpg]

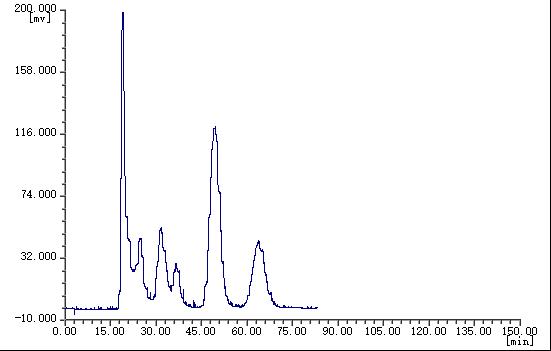

Supplement: Supplementary file 1 [file molecules-24-04496-s001.zip › supplementary material/medicinal materials/7.jpg]

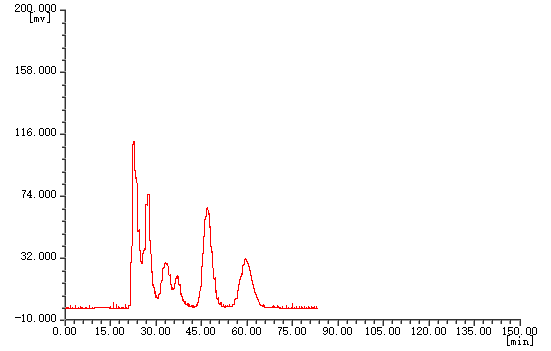

Supplement: Supplementary file 1 [file molecules-24-04496-s001.zip › supplementary material/medicinal materials/8.1.png]

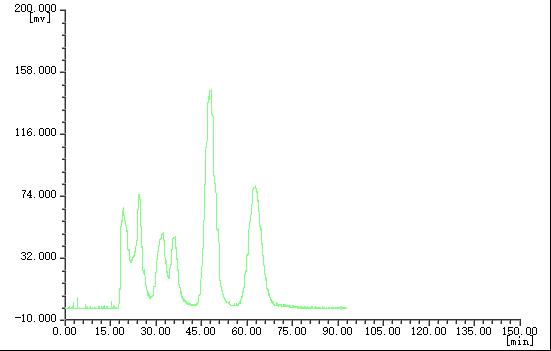

Supplement: Supplementary file 1 [file molecules-24-04496-s001.zip › supplementary material/medicinal materials/8.jpg]

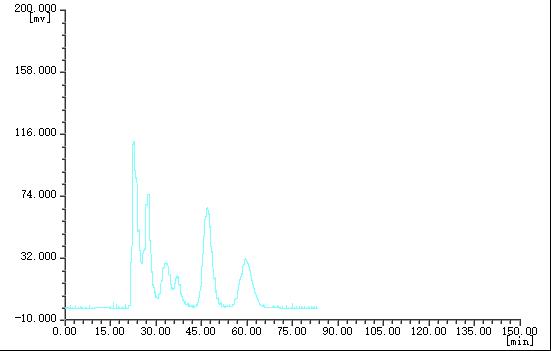

Supplement: Supplementary file 1 [file molecules-24-04496-s001.zip › supplementary material/medicinal materials/9.jpg]
